# Missouri Valley Veterinary Medical Association

**Published:** 1897-11

**Authors:** E. P. Schaffter

**Affiliations:** Secretary


Secretary.

### MISSOURI VALLEY VETERINARY MEDICAL ASSOCIATION.

THE Association convened in the lecture-room of the Kansas City Veterinary College on the evening of October 6, 1897, and was called to order by the President, Dr. S. L. Hunter. The following-named members were present: Drs. E. H. Biart, T. A. Bray, S. L. Brooking, John Ernst, R. H. Harrison, W. A. Heck, F. W. Hopkins, S. L. Hunter, G. A. Johnston, B. F. Kaupp, R. C. Moore, E. P. Schaffter, R. P. Steddon, S. Stewart, and J. B. Wright. A number of college students were also in attendance.

The President did not deliver an address, but gave in lieu thereof a financial statement of the affairs of the Association for the three years he served as Secretary and Treasurer. The statement showed the Association to have a small cash balance on hand and a considerable amount of unpaid dues still to be collected.

Dr. Brooking presented a very terse yet interesting paper on the subject of "Extra-uterine Pregnancy," in which he called attention to the frequency of its occurrence in the sow in contrast to its infrequency in the cow or mare. The Doctor had seen two extra-uterine fœtuses removed from the abdomen of one sow. The paper called forth an interesting discussion.

Dr. Kaupp presented a short paper on "Texas Fever," in which he called attention to the post-mortem lesions as seen on the slaughter-beds and noted the absence of many of the typical conditions seen in cases which died from the disease or had been killed shortly before they would have died, to study the disease lesions. The discussion of this paper was quite animated, and all the members—also some of the visitors—contributed points of interest.

Dr. Heck reported a very interesting and peculiar case of laceration of a muscular pillar of the diaphragm, with inflammation extending to the adjacent organs, in a horse. The case terminated in death. The Doctor noted the peculiar desire of the horse to lie in a wallow of mud in a sterno-ventral position with forelimbs flexed under the chest like the cow.

The Association passed a resolution instructing the Secretary to urge the U. S. V. M. A. to hold its next annual meeting at Omaha, Neb., and pledging its good offices in helping to make that meeting interesting and profitable.

Dr. Stewart kindly favored the Association with a talk on the proceedings of the U. S. V. M. A. meeting at Nashville, Tenn., which was listened to with great interest by all.

The Association adjourned to meet December 8, 1897, in Kansas City Veterinary College.

E. P. SCHAFFTER, V.S.,
Secretary.

